# Learning Extremal Representations with Deep Archetypal Analysis

**DOI:** 10.1007/s11263-020-01390-3

**Published:** 2020-12-23

**Authors:** Sebastian Mathias Keller, Maxim Samarin, Fabricio Arend Torres, Mario Wieser, Volker Roth

**Affiliations:** Department of Mathematics and Computer Science, University of Basel, Spiegelgasse 1, 4051 Basel, Switzerland

**Keywords:** Dimensionality reduction, Archetypal analysis, Deep variational information bottleneck, Generative modeling, Sentiment analysis, Chemical autoencoder

## Abstract

Archetypes represent extreme manifestations of a population with respect to specific characteristic traits or features. In linear feature space, archetypes approximate the data convex hull allowing all data points to be expressed as convex mixtures of archetypes. As mixing of archetypes is performed directly on the input data, linear Archetypal Analysis requires additivity of the input, which is a strong assumption unlikely to hold e.g. in case of image data. To address this problem, we propose learning an appropriate *latent* feature space while simultaneously identifying suitable archetypes. We thus introduce a generative formulation of the linear archetype model, parameterized by neural networks. By introducing the distance-dependent archetype loss, the linear archetype model can be integrated into the latent space of a deep variational information bottleneck and an optimal representation, together with the archetypes, can be learned end-to-end. Moreover, the information bottleneck framework allows for a natural incorporation of arbitrarily complex side information during training. As a consequence, learned archetypes become easily interpretable as they derive their meaning directly from the included side information. Applicability of the proposed method is demonstrated by exploring archetypes of female facial expressions while using multi-rater based emotion scores of these expressions as side information. A second application illustrates the exploration of the chemical space of small organic molecules. By using different kinds of side information we demonstrate how identified archetypes, along with their interpretation, largely depend on the side information provided.

**Supplementary Information:**

The online version contains supplementary material available at 10.1007/s11263-020-01390-3.

## Introduction

**Fig. 1:**
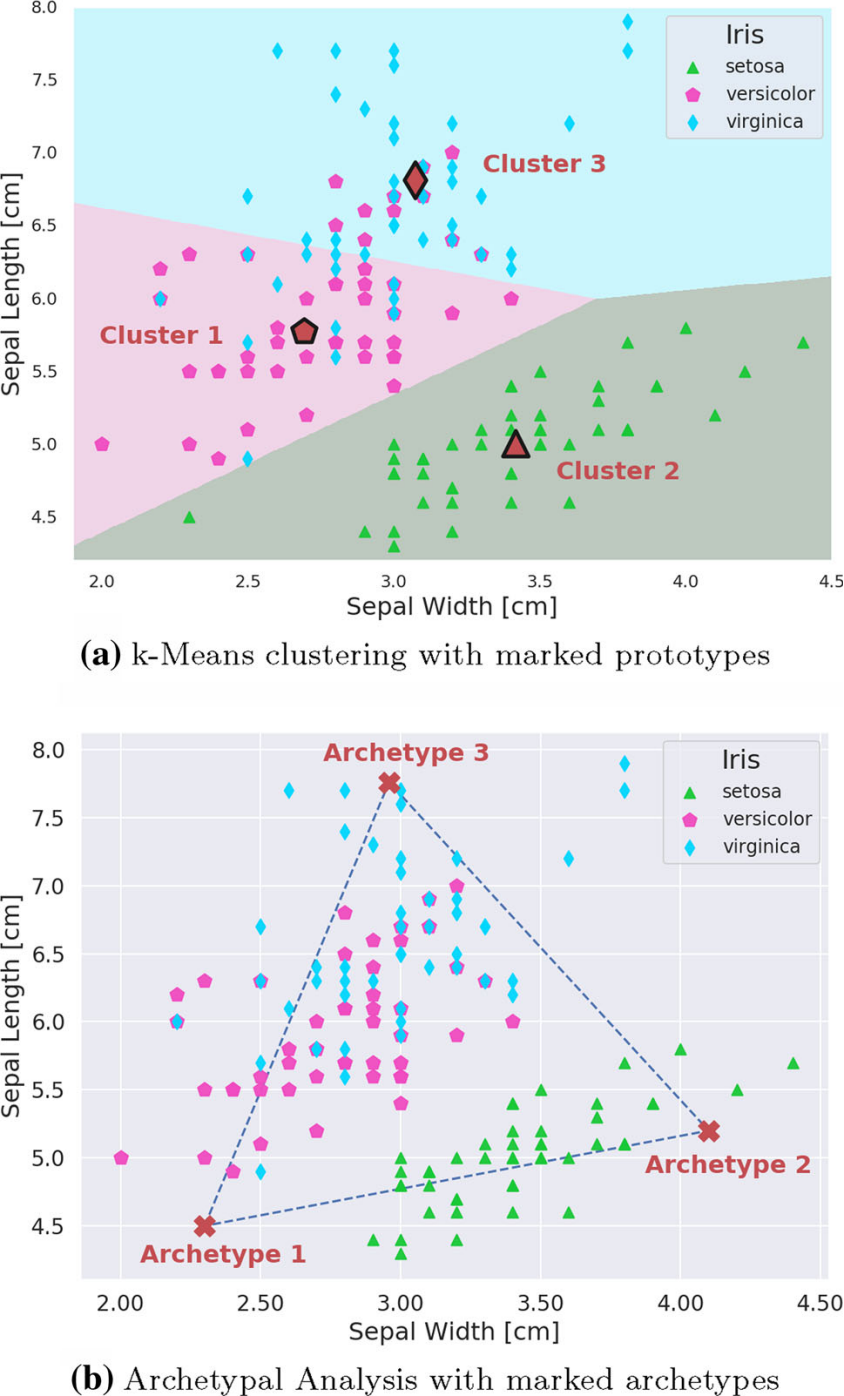
Result of a clustering procedure as well as an Archetypal Analysis, performed on the Iris data set. For clustering, the k-means algorithm was used, which is an unsupervised clustering algorithm identifying the average representatives of a data set, i.e. the cluster-centroids or prototypes. Archetypal Analysis on the other hand, seeks to identify extremes in the data set with the goal to represent individual data points as weighted mixtures of these extreme points, the so-called archetypes

Colloquially, both the words “archetype” and “prototype” describe templates or original patterns from which all later forms are developed. However, the concept of a prototype is more common in machine learning and for example encountered as cluster-centroids in classification, where a query point *x* is assigned to the class of the closest prototype. In an appropriate feature space such a prototype is a typical representative of its class, sharing all traits of the class members, ideally in equal proportion. By contrast, archetypes are characterized as being *extreme points* of the data, such that the complete data set can be well represented as a convex mixture of these extremes or archetypes. Archetypes thus form a polytope approximating the data convex hull. Based on the historic Iris flower data set (Anderson 1935; Fisher 1936), Fig. 1 illustrates the different perspectives both approaches provide in exploring the data. In Fig. 1a the cluster means as well as the decision boundaries in a 2-dimensional feature space are shown. The clustering was calculated using the k-Means algorithm. Each cluster mean is an *average* representative of its respective class, the aforementioned prototype. According to this clustering, the prototypical *Iris virginica* has a sepal width of 3.1 cm and a sepal length of 6.8 cm. On the other hand, Fig. 1b shows the positions of the three archetypal Iris flowers, which represent *extreme* manifestations of the Iris species of the respective classes. The archetypal *Iris virginica* has a sepal width of 3.0 cm and a sepal length of 7.8 cm. All flowers within the simplex are characterized as convex mixtures of these archetypes. As flowers *outside* of that simplex will also be described as convex mixtures, the linear archetype model will approximate their location in feature space by normal projections onto the simplex’ surface. With an increasing number of archetypes the approximation of the data convex hull will improve but interpretation of the individual archetypes might become more difficult. In general, a clustering approach is more natural if the existence of a cluster structure can be presumed. Otherwise, Archetypal Analysis might offer an interesting perspective for exploratory data analysis.

## Exploring Data Sets Through Archetypes

Archetypal analysis (AA) was first proposed by Cutler and Breiman (1994). It is a linear procedure where archetypes are selected by minimizing the squared error in representing each individual data point as a mixture of archetypes. Identifying the archetypes involves the minimization of a non-linear least squares loss.

### Archetypal Analysis

Linear AA is a form of non-negative matrix factorization where a matrix $X\in \mathbb {R}^{n\times p}$ of *n* data vectors is approximated as $X\approx ABX = AZ$ with $A\in \mathbb {R}^{n\times k}$, $B\in \mathbb {R}^{k\times n}$, and usually $k < \min \{n,p\}$. The so-called *archetype matrix* $Z\in \mathbb {R}^{k\times p}$ contains the *k* archetypes $\mathbf {z}_1,\ldots ,\mathbf {z}_j,\ldots , \mathbf {z}_k$ with the model being subject to the following constraints:

1 $$\begin{aligned} a_{ij} \ge 0 \wedge \sum _{j=1}^{k} a_{ij} = 1, \quad b_{ji} \ge 0 \wedge \sum _{i=1}^{n} b_{ji} = 1 \end{aligned}$$

Constraining the entries of *A* and *B* to be non-negative and demanding that both weight matrices are row stochastic implies a representation of the data vectors $\mathbf {x}_{i=1 \ldots n}$ as a weighted sum of the rows of *Z* while simultaneously representing the archetypes $\mathbf {z}_{j=1 \ldots k}$ themselves as a weighted sum of the *n* data vectors in *X*:

2 $$\begin{aligned} \mathbf {x}_i \approx \sum _{j=1}^{k} a_{ij} \mathbf {z}_j = \mathbf {a}_i Z, \quad \mathbf {z}_j = \sum _{i=1}^{n} b_{ji} \mathbf {x}_i = \mathbf {b}_j X \end{aligned}$$

Due to the constraints on *A* and *B* in Eq. 1 both the representation of $\mathbf {x}_i$ and $\mathbf {z}_j$ in Eq. 2 are *convex* combinations. Therefore the archetypes approximate the data convex hull and increasing the number *k* of archetypes improves this approximation. The central problem of AA is finding the weight matrices *A* and *B* for a given data matrix *X* and a given number *k* of archetypes. The non-linear optimization problem consists in minimizing the following residual sum of squares:

3 $$\begin{aligned} RSS(k)= & {} \min _{\mathbf {A},\mathbf {B}} \left\Vert \mathbf {X}-\mathbf {ABX} \right\Vert ^2 \end{aligned}$$

4 $$\begin{aligned}= & {} \min _{a,b} \sum _{l=1}^n \left\Vert \mathbf {x}_l - \sum _{j=1}^k a_{ij} \sum _{i=1}^n b_{ji} \mathbf {x}_i \right\Vert ^2 \end{aligned}$$

In their original publication, Cutler and Breiman (1994) propose an alternating least squares approach for finding the archetypes: After a random initialization of the *b*’s, Eq. 4 is solved for the *a*’s. Then, given the *a*’s, Eq. 4 is solved for the *b*’s. This alternating optimization, which provably converges towards a local minimum, can be implemented using common solvers for quadratic programming. Using an active set algorithm, together with smarter initialization strategies, higher convergence rates are achieved by the archetype algorithm proposed by Bauckhage and Thurau (2009). The *Rapid Archetypal Analysis* algorithm by Bauckhage et al. (2015) is based on a greedy Frank-Wolfe procedure and avoids, unlike the previously mentioned algorithms, the rather costly quadratic optimization routines. The example shown in Fig. 1b was calculated based on our own implementation of this algorithm, which is—to our knowledge—the most efficient algorithm for solving the linear archetype problem available today.

A probabilistic formulation of linear AA is provided by Seth and Eugster (2016) where it is observed that AA follows a simplex latent variable model and normal observation model. The generative process for the observations $\mathbf {x}_i$ in the presence of *k* archetypes with archetype weights $\mathbf{a} _i$ is given by

5 $$\begin{aligned} \mathbf {a}_i \sim \text {Dir}_k\left( \varvec{\alpha }\right) \quad \wedge \quad \mathbf {x}_i \sim \mathcal {N}\left( \mathbf {a}_iZ,\,\epsilon ^2 \mathbf {I}\right) , \end{aligned}$$

with uniform concentration parameters $\alpha _j = \alpha$ for all *j*, and weights summing up to $\Vert \mathbf {a}_i\Vert _1=1$. That is, the observations $\mathbf {x}_i$ are distributed according to isotropic Gaussians with means $\varvec{\mu }_i=\mathbf {a}_iZ$ and variance $\epsilon ^2$.

### A Biological Motivation for Archetypal Analysis

**Fig. 2:**
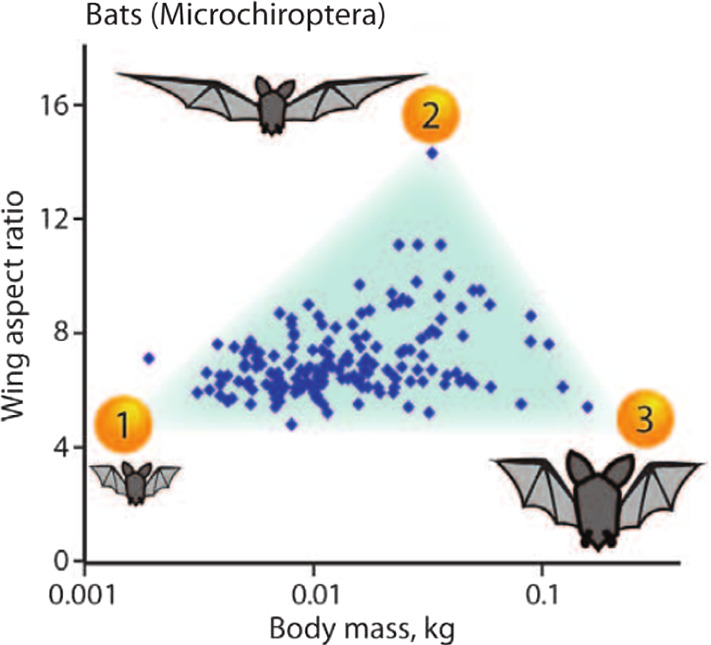
Phenospace of different species of Microchiroptera. The dominant food habit of each species, and thereby the ability to procure this food source, is linked to the morphology of the animals, e.g. a higher Wing Aspect Ratio corresponds with the greater aerodynamic efficiency needed to chase high flying insects. Archetypes are extreme types, optimized to perform a single task. Proximity of a species to an archetype quantifies the level of adaptation this species has undergone with respect to the optimization objective or task. Reprinted from Shoval et al. (2012) with permission

Conceptionally, the motivation for Archetypal Analysis is purely statistical but the method itself always implied the possibility of interpretations with a more *evolutionary flavour*. By representing an individual data point as a mixture of *pure types* or *archetypes*, a natural link to the evolutionary development of biological systems is implicitly established. The publication by Shoval et al. (2012) entitled ’Evolutionary Trade-Offs, Pareto Optimality, and the Geometry of Phenotype Space’ made this connection explicit, providing a theoretical foundation of the ’archetype concept’. In general, evolutionary processes are multi-objective optimization problems and as such subject to unavoidable trade-offs: If multiple tasks need to be performed, no (biological) system can be optimal at all tasks at once. Examples of such trade-offs include those between longevity and fecundity in Drosophila melanogaster where long-lived flies show decreased fecundity (Djawdan et al. 1996) or predators that evolve to be fast runners but eventually have to trade-off their ability to subdue large or strong prey, e.g. cheetah versus lion (Garland 2014). Such evolutionary trade-offs are known to affect the range of phenotypes found in nature (Tendler et al. 2015). In Shoval et al. (2012) it is argued that best-trade-off phenotypes are weighted averages of archetypes while archetypes themselves are phenotypes specialized at performing a *single* task optimally. An example of an evolutionary trade-off in the space of traits (or phenospace) for different species of bats (Microchiroptera) is shown in Fig. 2. Based on a study of bat wings by Norberg et al. (1987), each species is represented in a two-dimensional space where the axis depict Body Mass and Wing Aspect Ratio. The latter is the square of the wingspan divided by the wing area.

Table 1 gives an account of the task the archetypes indicated in Fig. 2 have evolved to performing optimally. The trade-off situation can be interpreted using Pareto optimality theory (Steuer 1986), which was recently used in biology to study trade-offs in evolution (Schuetz et al. 2012; El Samad et al. 2005). All phenotypes that have evolved over time lie within a restricted part of the phenospace, the so-called Pareto front, which is the set of phenotypes that cannot be improved at all tasks simultaneously. If there were a phenotype being better at all tasks than a second phenotype, then the latter would be eliminated over time by natural selection. Consequently phenotypes on the Pareto front are the best possible compromise between the different requirements or tasks.

**Table 1:** Inferred specialization of the archetypal species of Microchiroptera indicated in Fig. 2

Archetype	Phenotype	Specialization
1	Low aspect ratio, small body	Hunting small insects near vegetation
2	High aspect ratio, medium body	Hunting high flying large insects
3	Low aspect ratio, large body	Hunting animals near vegetation

From an evolutionary perspective, the phenotype is a consequence of the specialization, for details see (Shoval et al. 2012)

## Related Work

Linear “Archetypal Analysis” (AA) was first proposed by Cutler and Breiman (1994). Since its conception, AA has known several advancements on the *algorithmic side*: In Stone and Cutler (1996) the authors propose an archetype model able to identify archetypes in space *and* time, named “Archetypal Analysis of spatio-temporal dynamics”. A similar problem is addressed in “Moving archetypes” by Cutler and Stone (1997). Model selection is the topic of Prabhakaran et al. (2012), where the authors are concerned with the optimal number of archetypes needed to characterize a given data set. An extension of the original Archetypal Analysis model to non-linear kernel Archetypal Analysis is proposed by Bauckhage and Manshaei (2014); Mørup and Hansen (2012). In Kaufmann et al. (2015), the authors use a copula based approach to make AA independent of strictly monotone transformations of the input data. The reasoning is that such transformations should in general not influence which points are identified as archetypes. Algorithmic improvements by adapting a Frank–Wolfe type algorithm to speed-up the calculation of archetypes are made by Bauckhage et al. (2015). A probabilistic version of Archetypal Analysis was introduced by Seth and Eugster (2016), lifting the restriction of Archetypal Analysis to real–valued data and instead allowing other observation types such as integers, binary, and probability vectors as input. Efficient “coresets for Archetypal Analysis” are proposed by Mair and Brefeld (2019) in order to reduce the high computational cost due to the additional convexity-preserving constraints when identifying archetypes.

Although AA did not prevail as a commodity tool for pattern analysis, several applications have used it very successfully. In H P Chan et al. (2003), AA is used to analyse galaxy spectra which are viewed as weighted superpositions of the emissions from stellar populations, nebular emissions and nuclear activity. For the human genotype data studied by Huggins et al. (2007), inferred archetypes are interpreted as representative populations for the measured genotypes. In computer vision, AA has for example been used by Bauckhage and Thurau (2009) to find archetypal images in large image collections or by Canhasi and Kononenko (2015) to perform the analogous task for large document collections. In combination with deep learning, archetypal style analysis (Wynen et al. 2018) applies AA to learned image representations in order to realize artistic style manipulations.

Our work is based on the variational autoencoder model (VAE), arguably one of the most popular representatives of the class of “Deep Latent Variable Models”. VAEs were introduced by Kingma and Welling (2013); Rezende et al. (2014) and use an inference network to perform a variational approximation of the posterior distribution of the latent variables. Important work in this direction include Kingma et al. (2014); Rezende and Mohamed (2015) and Jang et al. (2017). More recently, Alemi et al. (2016) have discovered a close connection between VAE models and the Information Bottleneck principle (Tishby et al. 2000). Here, the Deep Variational Information Bottleneck (DVIB) is a VAE where not the input *X* is reconstructed (i.e. decoded) but rather a datum *Y*, about which *X* is known to contain information. Subsequently, the DVIB has been extended in multiple directions such as sparsity (Wieczorek et al. 2018), causality (Parbhoo et al. 2020) or invariant subspace learning (Wieser et al. 2020).

Akin to our work, *AAnet* is a model proposed by van Dijk et al. (2019) as an extension of linear Archetypal Analysis on the basis of standard, i.e. non-variational, autoencoders. In their work two regularization terms, applied to an intermediate representation, provide the latent archetypal convex representation of a non-linear transformation of the input. In contrast to our work, which is based on probabilistic generative models (VAE, DVIB), *AAnet* attempts to emulate the generative process by adding noise to the latent representation during training. Further, no side information is incorporated which can—and in our opinion should—be used to constrain potentially *over-flexible* neural networks and guide the optimisation process towards learning a meaningful representation. The work presented here is an extension of a conference paper by Keller et al. (2019). The extension highlights the wide scope of application of the proposed method by including extensive experiments on “Archetypes in Image-based Sentiment Analysis”. Furthermore, detailed discussions of the methodology have been added, in particular the sections on “Selecting the Number of Archetypes” and “The Necessity for Side Information”.

## Present Work

Archetypal analysis, as proposed by Cutler and Breiman (1994), is a linear method and cannot integrate any additional information about the data, e.g. labels, that might be available. Furthermore, the feature space in which AA is performed is spanned by features that had to be selected by the user based on prior knowledge. In the present work an extension of the original model is proposed such that appropriate representations can be learned end-to-end, side information can be incorporated to help learn these representations and non-linear relationships between features can be accounted for.

### Deep Variational Information Bottleneck

We propose a model to generalise linear AA to the non-linear case based on the Deep Variational Information Bottleneck framework since it allows to incorporate side information *Y* by design and is known to be equivalent to the VAE in the case of $Y=X$, as shown in Alemi et al. (2016). In contrast to the data matrix *X* in linear AA, a non-linear transformation *f*(*X*) giving rise to a latent representation $T\in \mathbb {R}^d$ of the data suitable for (non-linear) Archetypal Analysis is considered. I.e. the latent representation *T* takes the role of the data *X* in the previous treatment.

The DVIB combines the information bottleneck (IB) with the VAE approach (Tishby et al. 2000; Kingma and Welling 2013). The objective of the IB method is to find a random variable *T* which, while compressing a given random vector *X*, preserves as much information about a second given random vector *Y*. The objective function of the IB is as follows

6 $$\begin{aligned} \text{ min}_{p\left( \mathbf {t}|\mathbf {x}\right) } I(X;T) - \lambda I(T;Y), \end{aligned}$$

where $\lambda$ is a Lagrange multiplier and *I* denotes the mutual information. Assuming the IB Markov chain $T-X-Y$ and a parametric form of Eq. 6 with parametric conditionals $p_\phi (\mathbf {t}|\mathbf {x})$ and $p_\theta (\mathbf {y}|\mathbf {t})$, Eq. 6 is written as

7 $$\begin{aligned} \max _{\phi ,\theta } -I_{\phi }\left( \mathbf {t};\mathbf {x}\right) + \lambda I_{\phi ,\theta }\left( \mathbf {t};\mathbf {y}\right) . \end{aligned}$$

As derived in Wieczorek et al. (2018), the two terms in Eq. 7 have the following forms:

8 $$\begin{aligned} \begin{aligned} I_{\phi }\left( \mathbf {t};\mathbf {x}\right)&=\ D_{KL}\left( p(\mathbf {t}|\mathbf {x}) p(\mathbf {x}) \Vert p(\mathbf {t}) p(\mathbf {x})\right) \\&=\ \int p(\mathbf {t},\mathbf {x}) \log p_\phi (\mathbf {t}|\mathbf {x}) \mathrm {d}\mathbf {x}\,\mathrm {d}\mathbf {t}\\&\quad - \int p(\mathbf {x}|\mathbf {t}) p(\mathbf {t}) \log p(\mathbf {t}) \mathrm {d}\mathbf {x}\,\mathrm {d}\mathbf {t}\\&=\ \int p_\phi (\mathbf {t}|\mathbf {x}) p(\mathbf {x}) \log \frac{p_\phi (\mathbf {t}|\mathbf {x})}{ p(\mathbf {t}) } \, \mathrm {d}\mathbf {x}\,\mathrm {d}\mathbf {t}\\&=\ \mathbb {E}_{p(\mathbf {x})} D_{KL}\left( p_\phi (\mathbf {t}|\mathbf {x})\Vert p(\mathbf {t}) \right) \end{aligned} \end{aligned}$$

and

9 $$\begin{aligned} I_{\phi ,\theta }(\mathbf {t};\mathbf {y})= & {} \ D_{KL}\left( \left[ \int p(\mathbf {t}|\mathbf {y},\mathbf {x})p(\mathbf {y},\mathbf {x})\, \mathrm {d}\mathbf {x} \right] \Vert p(\mathbf {t}) p(\mathbf {y})\right) \nonumber \\&= \int p_\phi (\mathbf {t}|\mathbf {x},\mathbf {y}) p(\mathbf {x},\mathbf {y}) \log \frac{p_\theta (\mathbf {y}|\mathbf {t}) p(\mathbf {t})}{ p(\mathbf {t}) p(\mathbf {y})} \, \mathrm {d}\mathbf {t}\,\mathrm {d}\mathbf {x}\,\mathrm {d}\mathbf {y}\nonumber \\&=\ \mathbb {E}_{p(\mathbf {x},\mathbf {y})} \left[ \int p_\phi (\mathbf {t}|\mathbf {x},\mathbf {y}) \log p_\theta (\mathbf {y}|\mathbf {t}) \,\mathrm {d}\mathbf {t} \right] \nonumber \\&- \mathbb {E}_{p(\mathbf {x},\mathbf {y})} \left[ \log p(\mathbf {y}) \int p_\phi (\mathbf {t}|\mathbf {x},\mathbf {y}) \,\mathrm {d}\mathbf {t}\right] \nonumber \\\ge & {} \mathbb {E}_{p(\mathbf {x},\mathbf {y})} \mathbb {E}_{p_\phi (\mathbf {t}|\mathbf {x})}\log p_\theta (\mathbf {y}|\mathbf {t}) + h(Y). \end{aligned}$$

Here $h(Y)=-\mathbb {E}_{p(\mathbf {y})}\log p(\mathbf {y})$ denotes the entropy of *Y* in the discrete case or the differential entropy in the continuous case. The models in Eqs. 8 and 9 can be viewed as the encoder and decoder, respectively. Assuming a standard prior of the form $p(\mathbf {t})=\mathcal {N}(\mathbf {t};0,I)$ and a Gaussian distribution for the posterior $p_\phi (\mathbf {t}|\mathbf {x})$, the KL divergence in Eq. 8 becomes a KL divergence between two Gaussian distributions which can be expressed in analytical form as in Kingma and Welling (2013). *I*(*T*; *X*) can then be estimated on mini-batches of size *m* as

10 $$\begin{aligned} I_\phi \left( \mathbf {t};\mathbf {x}\right) \approx \frac{1}{m} \sum _i D_{KL}\left( p_\phi \left( \mathbf {t}|\mathbf {x}_i\right) \Vert p(\mathbf {t}) \right) . \end{aligned}$$

As for the decoder, $\mathbb {E}_{p(\mathbf {x},\mathbf {y})} \mathbb {E}_{p_\phi (\mathbf {t}|\mathbf {x})}\log p_\theta (\mathbf {y}|\mathbf {t})$ in Eq. 9 is estimated using the reparametrisation trick proposed by Kingma and Welling (2013); Rezende et al. (2014):

11 $$\begin{aligned} \begin{aligned} I_{\phi ,\theta }(\mathbf {t};\mathbf {y})&= \mathbb {E}_{p(\mathbf {x},\mathbf {y})} \mathbb {E}_{\varvec{\varepsilon } \sim \mathcal {N}(0,I)} \sum _i \log p_{\theta }\left( \mathbf {y}_i|\mathbf {t}_i \right) \\&+\text{ const. } \end{aligned} \end{aligned}$$

with the reparametrisation

12 $$\begin{aligned} \mathbf {t}_i = \varvec{\mu }_{i}(\mathbf {x}) + \text {diag}\left( \varvec{\sigma }_i(\mathbf {x})\right) \varvec{\varepsilon }. \end{aligned}$$

As mentioned earlier, in the case of $Y=X$ the original VAE is retrieved (Alemi et al. 2016). In our applications, we would like to predict not only the side information *Y* but also reconstruct the input *X*. Similar to the approach proposed in Gomez-Bombarelli et al. (2018), we use an additional decoder branch to predict the reconstruction $\tilde{X}$. This extension requires an additional term $I_{\phi ,\psi }(\mathbf {t};\varvec{\tilde{\mathrm{x}}})$ in the objective function Eq. 7 and an additional Lagrange multiplier $\nu$. The mutual information estimate $I_{\phi ,\psi }(\mathbf {t};\varvec{\tilde{\mathrm{x}}})$ is obtained analogously to Eq. 11.

### Deep Archetypal Analysis

Deep Archetypal Analysis can then be formulated in the following way. For the sampling of $\mathbf {t}_i$ in Eq. 11 the probabilistic AA approach as in Eq. 5 can be used which leads to

13 $$\begin{aligned} \mathbf {t}_i \sim \mathcal {N}\left( \varvec{\mu }_i(\mathbf {x})=\mathbf {a}_i(\mathbf {x}) Z,\,\varvec{\sigma }_i^2(\mathbf {x}) \mathbf {I}\right) , \end{aligned}$$

where the mean $\varvec{\mu }_i$ given through $\mathbf {a}_i$ and variance $\varvec{\sigma }_i^2$ are non-linear transformations of the data point $\mathbf {x}_i$ learned by the encoder. We note that the means $\varvec{\mu }_i$ are convex combinations of weight vectors $\mathbf {a}_i$ and the archetypes $\mathbf {z}_{j=1..k}$ which in return are considered to be convex combinations of the means $\varvec{\mu }_{i=1..m}$ and weight vectors $\mathbf {b}_j$.^1^ By learning weight matrices $A\in \mathbb {R}^{m\times k}$ and $B\in \mathbb {R}^{k\times m}$ which are subject to the constraints formulated in Eq. 1 and parameterised by $\phi$, a non-linear transformation of data *X* is learned which drives the structure of the latent space to form archetypes whose convex combination yield the transformed data points. A major difference to linear AA is that for *deep AA* we cannot identify the positions of the archetypes $\mathbf {z}_j$ as there is no absolute frame of reference in latent space. We thus position *k* archetypes at the vertex points of a $(k-1)$-simplex and collect these *fixed* coordinates in the matrix $Z^{\text {fixed}}$. These requirements lead to an additional distance-dependent archetype loss of

14 $$\begin{aligned} \ell _{\text {AT}} = ||Z^{\text {fixed}}-BAZ^{\text {fixed}}||_2^2 = ||Z^{\text {fixed}}-Z^{\text {pred}}||_2^2, \end{aligned}$$

where $Z^\text {pred} = BAZ^{\text {fixed}}$ are the *predicted* archetype positions given the learned weight matrices *A* and *B*. For $Z^\text {pred}\approx Z^\text {fixed}$ the loss function $\ell _{\text {AT}}$ is minimized and the desired archetypal structure is achieved. The objective function of *deep AA* is then given by

15 $$\begin{aligned} \max _{\phi ,\theta } -I_{\phi }\left( \mathbf {t};\mathbf {x}\right) + \lambda I_{\phi ,\theta }\left( \mathbf {t};\mathbf {y}\right) + \nu I_{\phi ,\psi }\left( \mathbf {t};\varvec{\tilde{\mathrm{x}}}\right) -\ell _{\text {AT}}. \end{aligned}$$

**Fig. 3:**
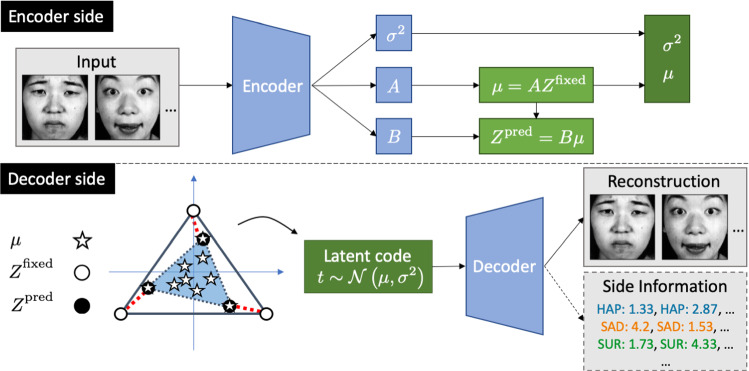
Illustration of the *deep AA* model. **Encoder side**: Learning weight matrices *A* and *B* allows to compute the archetype loss $\ell _{AT}$ in Eq. 14 and sample latent variables $\mathbf {t}$ as described in Eq. 13. The constraints on *A* and *B* in Eq. 1 are enforced by using softmax layers. **Decoder side**: $Z^\text {fixed}$ represent the fixed archetype positions in latent space while $Z^\text {pred}$ are given by the convex hull of the transformed data point means $\mu$ during training. Minimizing $\ell _{AT}$ corresponds to minimizing the red-dashed (pairwise) distances. The input is reconstructed from the latent variable $\mathbf {t}$. In the presence of side information, the latent representation allows to reproduce the side information *Y* as well as the input *X*

A visual illustration of *deep AA* is given in Fig. 3. The constraints on *A* and *B* can be guaranteed by using softmax layers and *deep AA* can be trained with a standard stochastic gradient descent technique such as Adam (Kingma and Ba 2014). Note that the model naturally allows to be relaxed to the VAE setting by omitting the side information term $\lambda I_{\phi ,\theta }(\mathbf {t};\mathbf {y})$ in Eq. 15.

### Selecting the Number of Archetypes

In the proposed model the dimension *d* of the latent space and the number of archetypes *k* are related through the equation $k=d+1$. The coordinates of the *k* archetypes coincide with the vertices of a regular *d*-simplex located on the unit sphere centered around the origin. Therefore, every vertex of the simplex has the same distance to the origin. Together with a spherical Gaussian prior $p(\mathbf {t})=\mathcal {N}(\mathbf {t};0,I)$, this geometric construct ensures that no latent space directions is preferred over any other. Thus, in the absence of prior knowledge, this agnostic setting makes all archetypes equally important.

In principal, decoupling *k* and *d* is a valid option. But by increasing the number of archetypes *k* in a latent space of fixed dimension, every data set can be explained in an increasingly trivial manner. The idea of Archetypal Analysis, however, is to tolerate some noise in the generative process and to approximate the convex hull with only a limited number of vertex points. Within the framework of a variational information bottleneck, choosing $k=d+1$ thus allows to identify the most compact latent code for a given data set. Consequently, model selection is performed by observing at which latent dimensionality the predictive mutual information, i.e. the reconstruction loss, saturates. In Sect. 5.3, we demonstrate the model selection process using a held-out test set in the experiments based on the QM9 data set of small organic molecules. Additionally, in Sect. 5.4, we explore an alternative prior conceptually closer to the original formulation of Archetypal Analysis.

### The Necessity for Side Information

The goal of deep AA is to identify meaningful archetypes in latent space which will subsequently enable an informed exploration of the given data set. The *meaning* of an archetype, and thereby the associated interpretation, can be improved by providing so-called side information, i.e. information *in addition* to the input data. For non-linear latent variable models parametrized by neural networks, an interpretation of the latent space structure—depending on the data set—is often difficult, as input dimensions can be mapped to arbitrarily complex non-linear curves in latent space. In general, more non-linearity leads to more flexibility in the mapping of an input onto its latent code, which in turn leads to more ambiguity when interpreting that latent code. Supplementing the training process with additional information— which we call *side information*—can facilitate the interpretation. Consequently, the function of the side information is that of a regularizer as it restricts the class of potential mappings. If the input datum is for example an image, additional information could simply be a scalar- or vector-valued label. Using richer side information, e.g. additional images, is of course possible. In more general terms, the fundamental idea is that information about what constitutes an *archetypal* representative might not be information that is readily present in the input *X* but dependent on—or even defined by—the side information. Taking a data set of car images as an example, what would be an *archetypal* car? Certainly, the overall size of a car would be a good candidate, such that smaller sports cars and larger pick-ups might be identified as archetypes. But introducing the fuel consumption of each car as side information would put sports cars and pick-ups closer together in latent space, as both car types often consume above average quantities of fuel. In this way, side information guides the learning of a latent representation which is informative with respect to exactly the side information provided. Consequently, whether a data point is identified as an archetype, is not an inherent property of the data alone, but rather a function of the side information made available during training. And the selection of appropriate side information can only be linked to the questions the user of a deep AA model is interested in answering.

## Experiments

### Archetypal Analysis: Dealing With Non-linearity

*Data generation.* For this experiment, data $\mathbf {X}\in \mathbb {R}^{n\times 8}$ is generated that is a convex mixture of *k* archetypes $\mathbf {Z}\in \mathbb {R}^{k\times 8}$ with $k\ll n$. The generative process for the datum $\mathbf {x_i}$ follows Eq. 5, where $\mathbf {a}_i$ is a stochastic weight vector denoting the fraction of each of the *k* archetypes $\mathbf {z}_j$ needed to represent the data point $\mathbf {x}_i$. A total of $n=10000$ data points is generated, of which $k=3$ are true archetypes. The variance is set to $\sigma ^2=0.05$ and the linear 3-dim data manifold is embedded in a $n=8$ dimensional space. Note that although linear and deep Archetypal Analysis is always performed on the full data set, only a fraction of that data is displayed when visualizing results.

**Fig. 4:**
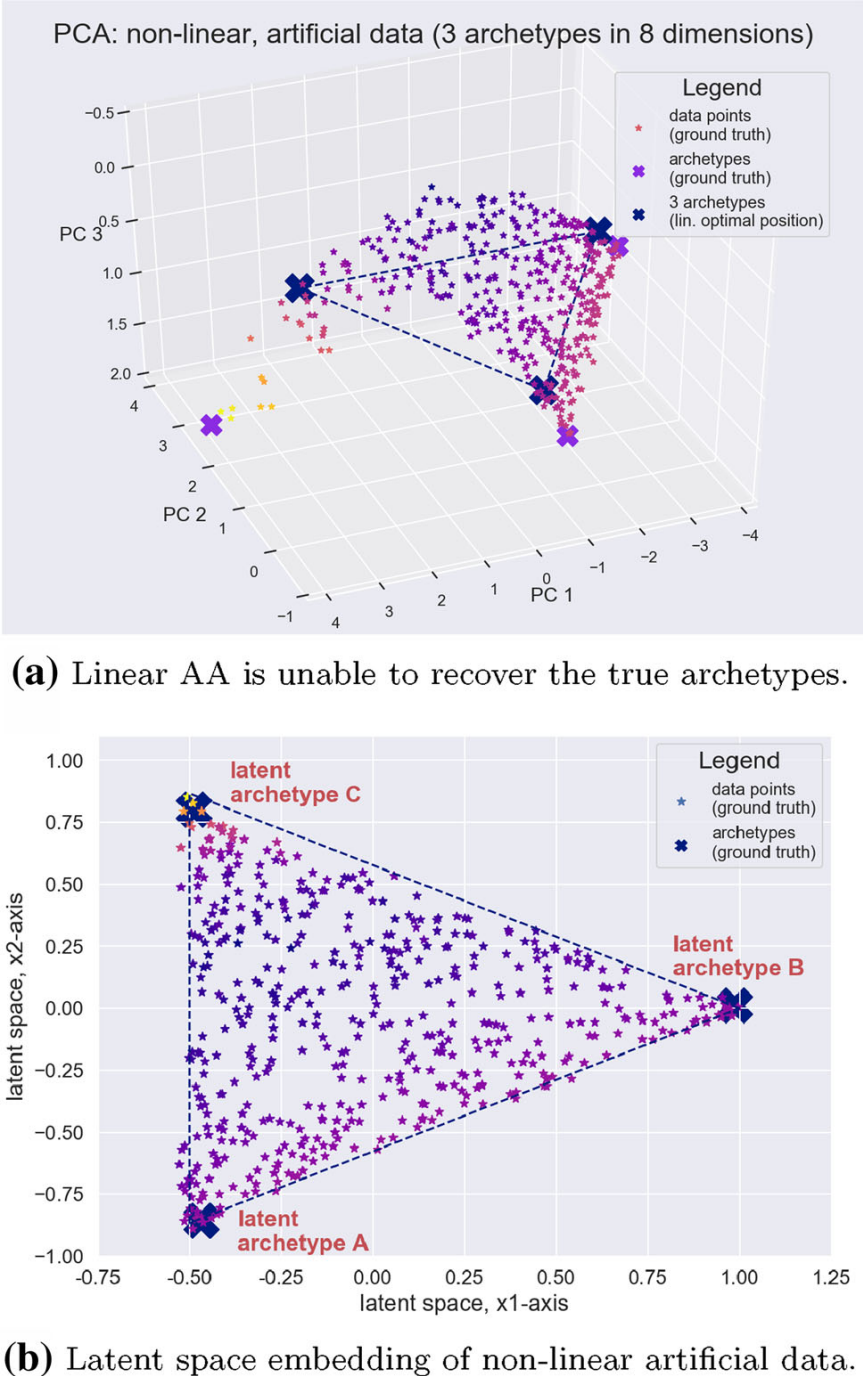
While linear Archetypal Analysis is in general unable to approximate the convex hull of a non-linear data set well, deep AA learns an appropriate latent representation where the ground truth archetypes can correctly be identifie

*Linear AA—non-linear data.* Data is generated as described above and an additional non-linearity is introduced by applying an exponential to one dimension of $\mathbf {X}$ which results in a curved 8-dimensional data manifold. Linear Archetypal Analysis is then performed using the efficient Frank-Wolfe procedure proposed by Bauckhage et al. (2015). For visualization, PCA is used to recover the original 3-dimensional data submanifold which is embedded in the 8-dimensional space. The first three principal components of the ground truth data are shown in Fig. 4a as well as the computed archetypes (connected by dashed lines). The positions of the computed archetypes occupy optimal positions according to the optimization problem in Eq. 3 but due to the non-linearity in the data it is impossible to recover the three ground truth archetypes.

*Deep AA—non-linear data.* For data that has been generated as described in the previous paragraph, a strictly monotone transformation in form of an exponentiation should in general *not* change which data points are identified as archetypes. But this is clearly the case for linear AA as it is unable to recover the true archetypes *after* a non-linearity has been applied. Using that same data to train the deep AA architecture presented in Fig. 3 generates the latent space structure shown in Fig. 4b, where the three archetypes A, B and C have been assigned to the appropriate vertices of the latent simplex. Moreover, the sequence of color stripes shown has been correctly mapped into the latent space. Within the latent space data points are again described as convex linear combinations of the latent archetypes. Latent data points can also be reconstructed in the original data space through the learned decoder network. The network architecture used for this experiment was a simple feedforward network (2 layered encoder and decoder), training for 20 epochs with a batch size of 100 and a learning rate of 0.001.

### Archetypes in Image-Based Sentiment Analysis

The Japanese Female Facial Expression (JAFFE) database was introduced by Lyons et al. (1998) and contains 213 images of 7 facial expressions (6 basic facial expressions + 1 neutral). The expressions are happiness, sadness, surprise, anger, disgust and fear. All expressions were posed by 10 Japanese female models. Each image has been rated on 6 emotion adjectives by 60 Japanese subjects on a 5 level scale (5-high, 1-low) and each image was then assigned a 6-dimensional vector of average ratings. For the following experiments the advice of the creator of the JAFFE data set was followed to exclude *fear* images and the *fear* adjective from the ratings, as the models were not believed to be good at posing fear. All experiments based on the JAFFE data set are performed on the following architecture^2^:

- **Encoder**:
  Input: image $$\mathbf {x}$$ $$(128\times 128)$$
  $$\rightarrow 3\times \Big [64\,\hbox {Conv}.\,(4\times 4) + \hbox {Max-Pool}. (2\times 2)\Big ]$$
  $$\rightarrow $$ Flatten + FC100
  $$\rightarrow $$ $$\mathbf {A}$$, $$\mathbf {B}$$, $$\sigma ^2$$
- **Decoder (Image Branch)**:
  Input: latent code $\mathbf {t}$
  $$\rightarrow $$ FC49
  $$\rightarrow $$ $$3\times \Big [64 \hbox {Conv. Transpose} (4\times 4)\Big ]$$
  $$\rightarrow $$ $$\hbox {Flatten} + \hbox {FC128} \times 128$$
  $$\rightarrow $$ $$\hbox {FC128}\times 128 \rightarrow 128\times 128 \hbox {reconstruction}~ \varvec{\tilde{\mathrm{x}}}$$
- **Decoder (Side Information Branch)**:
  Input: latent code $\mathbf {t}$
  $\rightarrow$ FC200-5 $\rightarrow$ side information $\tilde{\mathbf {y}}$

ReLU activations are used in-between layers and sigmoid activations for the image intensities. The different losses are weighted as follows: we multiplied the archetype loss by a factor of 80, the side information loss by 560, and the KL divergence by 40. In the setting where only two labels are considered, the weight for archetype loss is increased to 120. The network was trained for 5000 epochs with a mini-batch size of 50 and a learning rate of 0.0001. For training a NVIDIA TITAN X Pascal GPU was used, where a full training sessions lasted approximately 30 min.

#### JAFFE: Latent Space Structure

Emotions conveyed through facial expressions are a suitable case to demonstrate the interpretability of learned latent representation in deep AA. First, the existence of archetypes is plausible as there clearly are expressions that convey a maximum of a given emotion, i.e. a person can look extremely/maximally surprised.

**Fig. 5:**
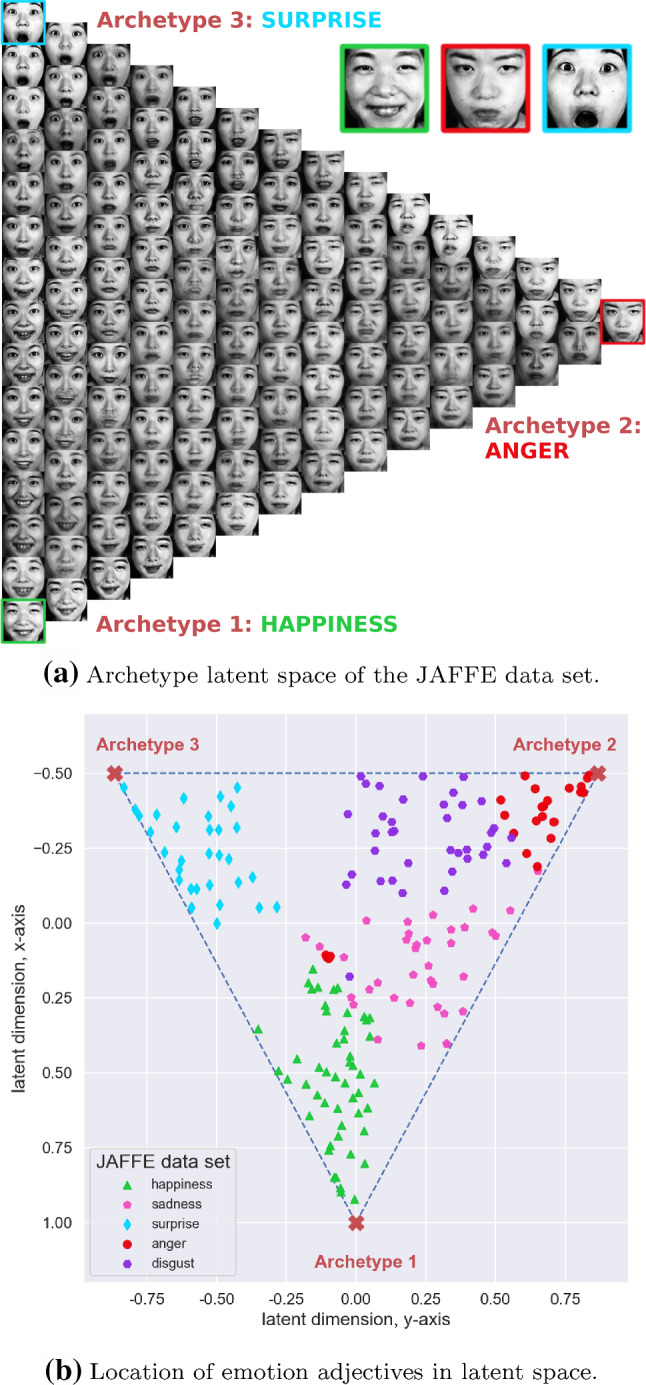
Deep AA with $k=3$ archetypes identifies sadness as a mixture mostly between happiness and anger while disgust lies between the archetypes for anger and surprise

Second, facial expressions change continuously without having a clearly defined cluster structure. Moreover, these expressions lend themselves to being interpreted as mixtures of basic (or archetypal) emotional expressions—a perspective also enforced by the averaged ratings for each image which are essentially weight vectors with respect to the archetypal emotional expressions. Figure 5a shows the learned archetypes “happiness”, “anger” and “surprise” while expressions linked to the emotion adjective “sadness” are identified as mixtures between archetype 1 (happiness) and archetype 2 (anger). Figure 5b shows the positions of the latent means where the color coding is based on the *argmax* of the emotion rating, which is a 5-dimensional vector. An analogous situation is found in case of “disgust”, which, according to deep AA, is a mixture between archetype 2 (anger) and archetype 3 (surprise). Towards the center of the simplex, expressions are located which share equal weights with respect to the archetypes and thus resemble a more “neutral” facial expression.

Section S1 of the supplement contains an additional experiment based on 40 independent bootstrap runs using the JAFFE data set. It highlights the stability of the inferred archetypes, where the predominant combination of inferred archetypes contains the emotions “surprise”, “happiness” and “anger”, as shown in Fig. 5b.

*Side Information for JAFFE.* The JAFFE data set contains facial expressions posed by 10 Japanese female models. Based solely on the visual information, i.e. disregarding the emotion scores, these images could meaningfully be grouped together in a variety of ways, e.g. head shape, hair style, identity of the model posing the expressions etc. The interpretability of archetypes, in general, rests on providing side information with respect to which the learned representation shall be informative.

The latent space shown in Fig. 8 has been learned while providing only the emotion ratings for “sadness” and “disgust”. This result illustrates how side information is shaping the structure of the learned latent representation: Comparing Fig. 8 with Fig. 5a, where the emotion ratings for “anger”, “surprise” and “sadness” were provided as side information during training, makes clear that archetypes are not necessarily a property of the data. The final structure of the latent space is determined to a large extend by the side information and thus by the intent of the user when selection which information to provide.

While it is obvious to learn typical emotion expressions in case of JAFFE, most applications are arguably more ambiguous. In Sect. 5.3, a chemical experiment is discussed, where each molecule can be described by a variety of properties. The side information introduced to the learning process will ultimately be the property the experimenter is interested in, and the learned representation will be informative with respect to that property.

#### JAFFE: Expressions As Weighted Mixtures

**Fig. 6:**
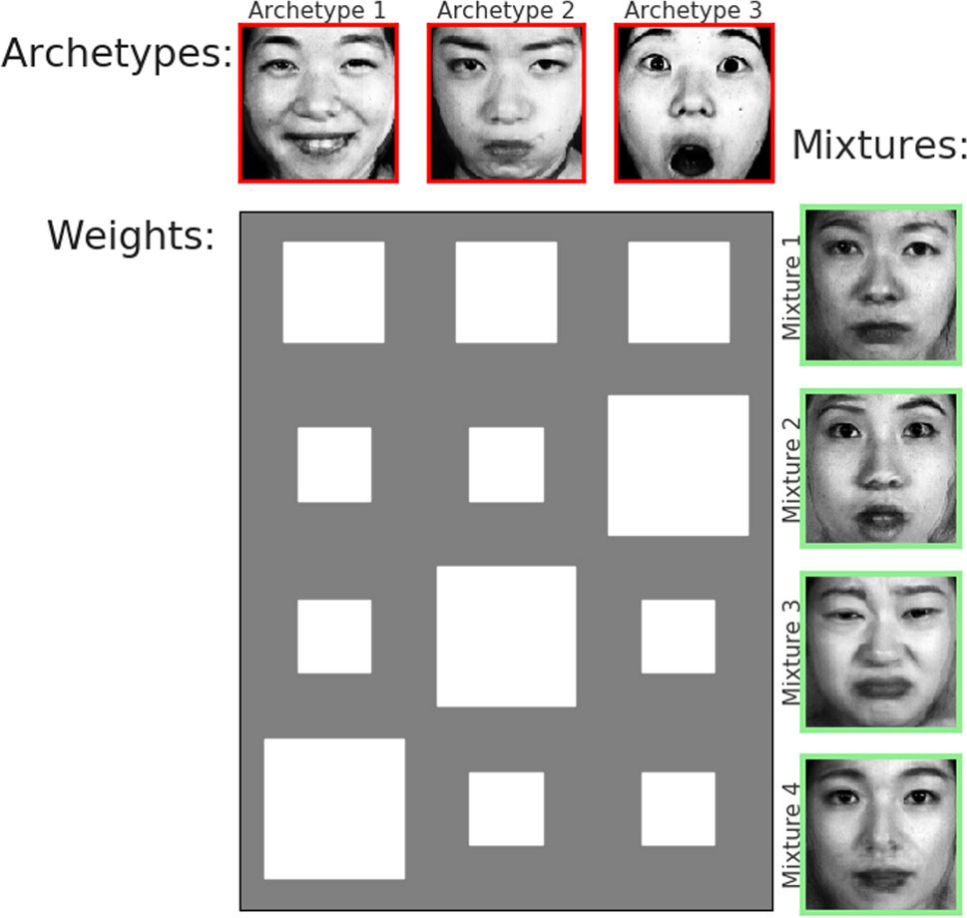
Knowing the archetypes allows for an informed exploration of the latent space by *not* directly sampling latent space coordinates but by specifying a desired mixture with respect to the known archetypes

One advantage of deep AA compared to the plain Variational Autoencoder (VAE) is a *globally* interpretable latent structure. All latent means $\mu _i$ will be mapped inside the convex region spanned by the archetypes. And as archetypes represent extremes of the data set which are present to some percentage in all data points, these percentages or weights can be used to explore the latent space in an *informed* fashion. This might be especially of advantage in case of higher-dimensional latent spaces. For example, the center of the simplex will always accommodate latent representations of input data that are considered *mean* samples of the data set. Moreover, directions within the simplex have meaning in the sense that when “walking” towards or away from a given archetype, the characteristics of that archetype will either be enforce or diminished in the decoded datum associated with the actual latent position. This is shown in the Hinton plot in Fig. 6 where mixture 1 is a mean sample, i.e. with equal archetype weights. Starting at this position and moving on a straight line into the direction of archetype 3 increases its influence while equally diminishing the influence of both archetypes 1 and 2. This results in mixture 2 which starts to look surprised, but not as extremely surprised as archetype 3. In the same fashion mixture 3 and 4 are the results of walking straight into the direction of archetypes 2 or 1 which results in a sad face (mixture 3) and a slightly happy facial expression (mixture 4).

#### JAFFE: Deep AA Versus VAE

**Fig. 7:**
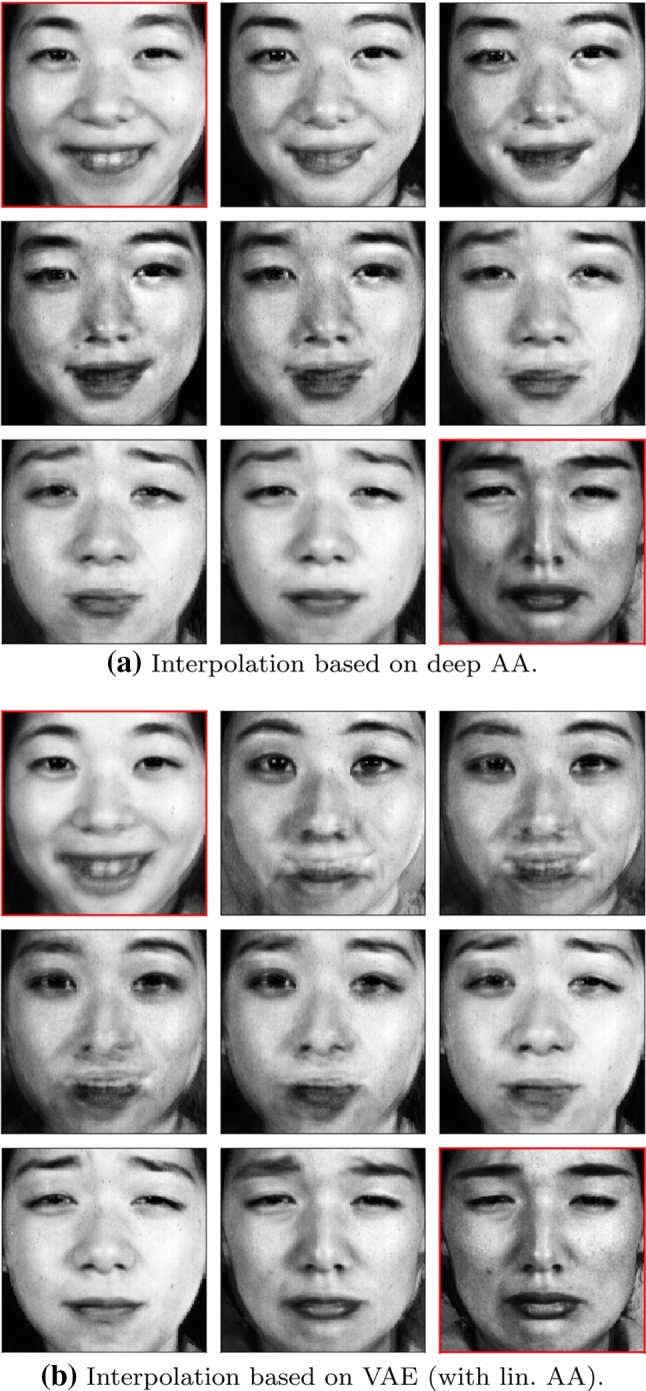
Interpolation between the two input images marked in red. The interpolation in the latent space of the deep AA model is qualitatively better compared to the VAE model as latent points are mapped more densely due to the simplex constraints

Deep AA is designed to be a model that simultaneously learns an appropriate representation and identifies meaningful latent archetypes. This model can be compared to a plain VAE where a latent space is learned first and subsequently linear AA is performed on that space in order to approximate the latent convex hull. Figure 7a shows the interpolation in the deep AA model between two images, neither of them archetypes, from “happy” to “sad”. Compared to Fig. 7b, which shows the same interpolation in a VAE model with subsequently performed linear AA, the interpolation based on deep AA gives a markedly better visual impression. In case of deep AA, this is explained by the fact that all data points are mapped into the simplex which ensures a relatively dense distribution of the latent means. On the other hand, the latent space of the VAE model has no hard geometrical restrictions and thus the distribution of the latent representatives will be less dense or even “patchy”, i.e. with larger empty areas in latent space. Especially with small data sets such as JAFFE, of which less than 200 images are used, interpolation quality might be strongly affected by the unboundedness of the latent space of VAE models.

**Fig. 8:**
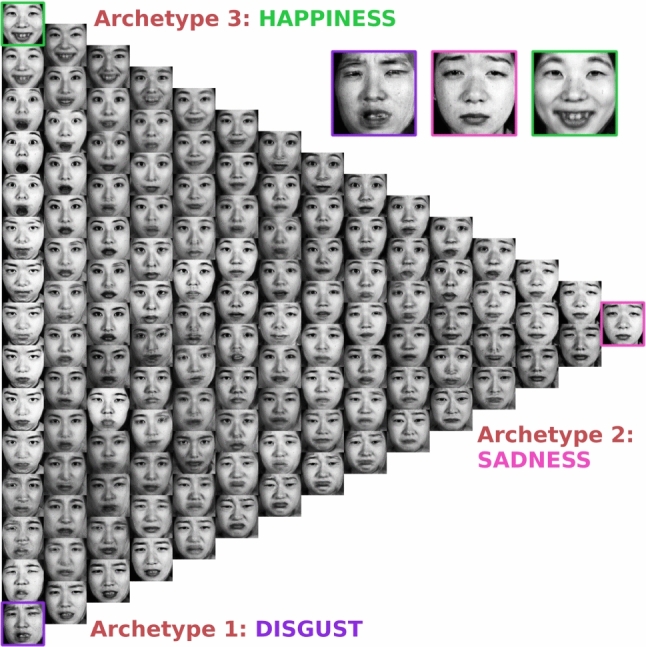
Latent structure of the JAFFE data set when trained on a subset of the side information containing only the emotion ratings for “sadness” and “disgust”

### The Chemical Universe Of Molecules

In the following section the application of deep AA to the domain of chemistry is explored. Starting with an initial set of chemical compounds, e.g. small organic molecules with cyclic cores (Visini et al. 2017), and iteratively applying a finite number of reactions, will eventually lead to a huge collection of molecules with extreme combinatorial complexity. But while the total number of all possible *small* organic molecules has been estimated to exceed $10^{60}$ (Kirkpatrick and Ellis 2004), even this number pales in comparison to the whole chemical universe of organic chemistry. In general, the efficient exploration of chemical spaces requires methods capable of learning meaningful representations and endowing these spaces with a globally interpretable structure. Prominent examples of chemistry data sets include the family of GDB-xx data sets (generic database), e.g. GDB-13 (Blum and Reymond 2009), which enumerates small organic molecules of up to 13 atoms, composed of the elements C, N, O, S and Cl, following simple chemical stability and synthetic feasibility rules. With more than 970 million structures, GDB-13 is the largest publicly available database of small organic molecule to date.

**Fig. 9:**
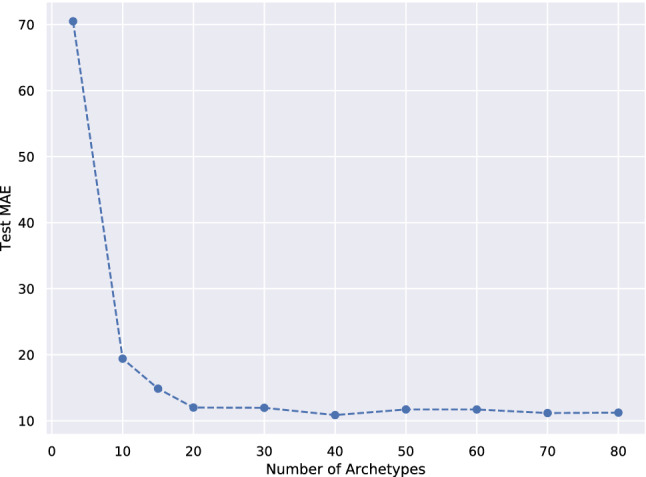
Model selection on the QM9 data set: Mean absolute error (reconstruction loss) vs. number of archetypes on the test set

**Fig. 10:**
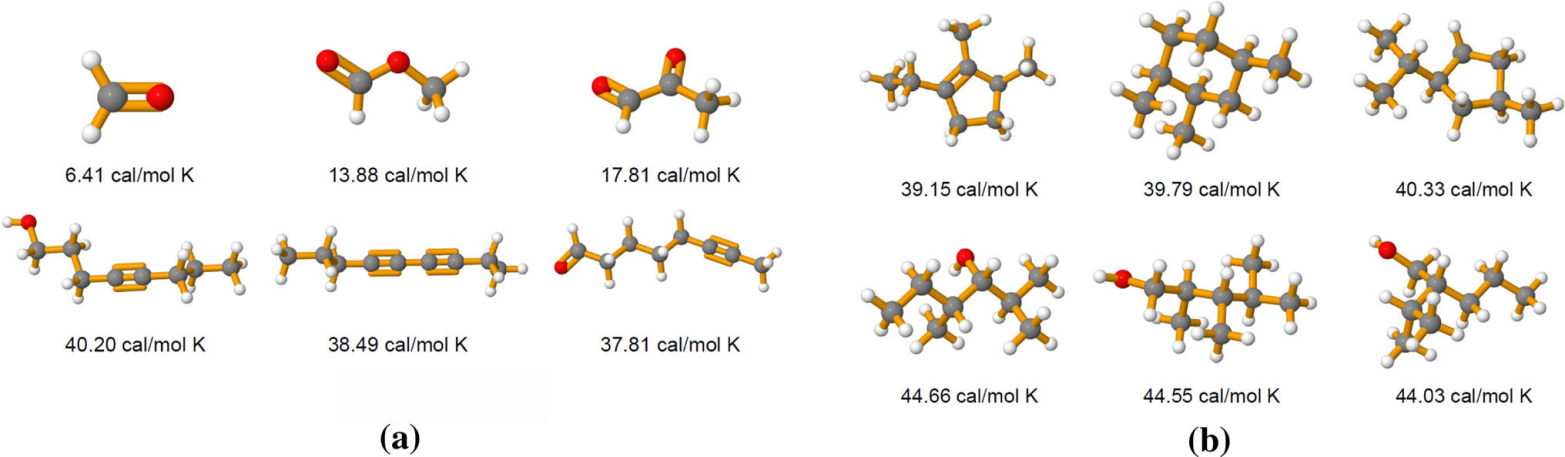
Both panels illustrate a comparison between archetypal molecules, where the underlying latent representation is informative with respect to the molecular property *heat capacity*. Each row contains the three molecules of the test set that have been mapped closest to a specific vertex of the latent simplex. Panel **a** compares archetypal *linear* molecules characterized by a short chain structure versus long chained molecules. Panel **b** compares archetypal molecules with similar masses but different geometric configuration, i.e. with and without a cyclic structure

*Exploring the Chemical Space.*

As discussed in Sect. 2.2, Archetypal Analysis lends itself to a distinctly evolutionary interpretation. Although this is certainly a more biological perspective, the basic principle is applicable to other fields. In chemistry, the principle of *evolutive abiogenesis* describes a process in which simple organic compounds increase in complexity (Miller 1953). In the following experiment a structured chemical space is learned using as side information the *heat capacity* $C_v$ which quantifies the amount of energy (in Joule) needed to increase 1 Mol of molecules by 1 K at constant volume. A high $C_v$ number is important e.g. in applications dealing with the storage of thermal energy (Cabeza et al. 2015). In the following, all experiments are based on the QM9 data set (Ramakrishnan et al. 2014; Ruddigkeit et al. 2012), which contains molecular structures and properties of 134k organic molecules. Each molecule is made up of nine or less atoms, i.e. C, O, N, or F, without counting hydrogen. The QM9 data set is based on ab-initio density functional theory (DFT) calculations.

**Fig. 11:**
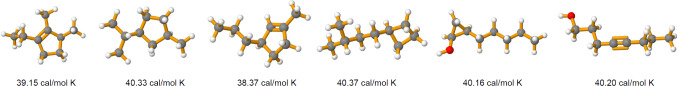
Interpolation between two archetypal molecules produced by deep AA. The labels display the heat capacity of each molecule. Here, only a single example is shown but similar results can be observed for other combinations of archetypes

*Experiment Setup.* A total of 204 features were extracted for every molecule using the Chemistry Development Kit (Steinbeck et al. 2003). The neural architecture used has 3 hidden FC layers with 1024, 512 and 256 neurons, respectively, and ReLU activation functions. For all experiments, the model was trained in a *supervised* fashion by reconstructing the molecules and the side information simultaneously. In *Experiment 1*, model selection was performed by continuously increasing the number of latent dimensions. Based on the knee of the mean absolute error (MAE), the appropriate number of latent archetypes was selected. In *Experiments 2 and 3*, the number of latent dimensions was fixed to 19, corresponding to the optimal number of 20 archetypes from the model selection procedure. During training, the Lagrange multiplier $\lambda$ was steadily increased by increments of 1.01 every 500 iterations. For training, the Adam optimizer (Kingma and Ba 2014) was used, with an initial learning rate of 0.01. A learning rate decay was introduced, with an exponential decay of 0.95 every 10k iterations. The batch size was 2048 and the model was trained for a total of 350 k iterations. The data set is divided in training and test set with a 90%/10% split. For visualization, the 3-dimensional molecular representations haven been created with Jmol (2019).

*Experiment 1: Model Selection.* The mean absolute error is assessed while varying the number of archetypes. The result is shown in Fig. 9. Model selection is performed by observing for which number of archetypes the MAE starts to converge. The knee of this curve is used to select the optimal number of archetypes, which is 20. Obviously, if the number of archetypes is smaller, it becomes more difficult to reconstruct the data. This is explained by the fact that there exists a large number of molecules with *very similar* heat capacities but at the same time *distinctly different* geometric configurations. As a consequence, molecules with different configurations are mapped to archetypes with the similar heat capacity, making it hard to resolve the many–to–one mapping in the latent space.

**Fig. 12:**
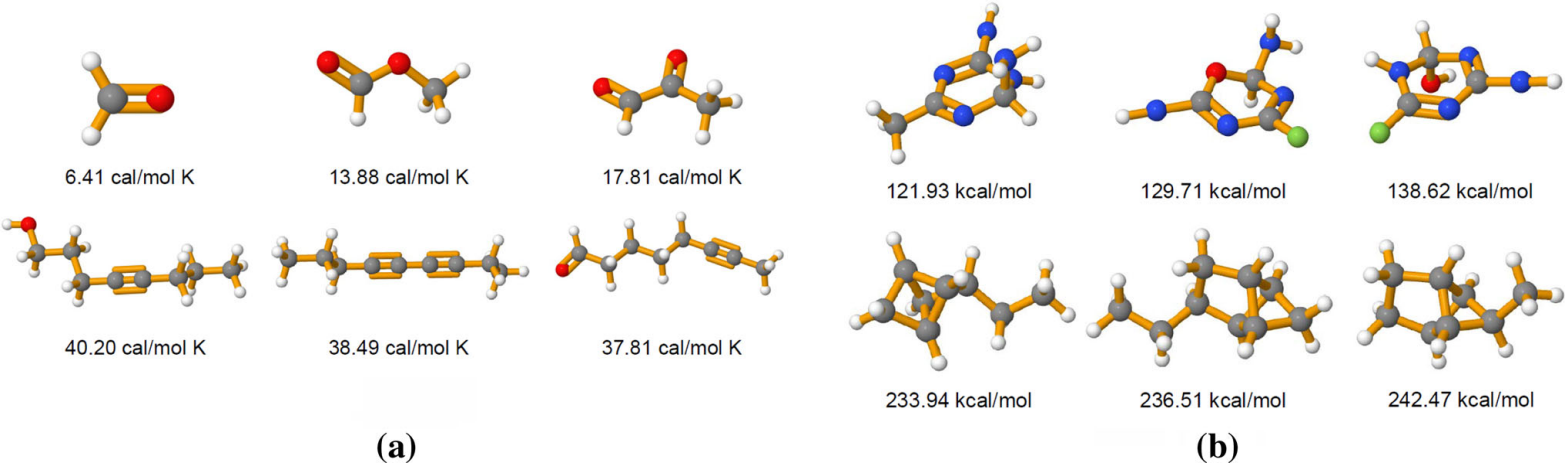
Panels **a** and **b** compare archetypal molecules identified using different side information: Here, the labels correspond to the heat capacity (panel a) and the band gap energy (panel b). The rows contain the three molecules of the test set closest to the given archetype

*Experiment 2: Archetypal Molecules.* Archetypal molecules are identified along with the heat capacities associated with them. A fixed number of 20 archetypes is used for optimal exploration-exploitation trade-off, in accordance with the model selection discussed in the previous section. In chemistry, the heat capacity at constant volume is defined as $C_v = \dfrac{d\epsilon }{dT} \bigm |_{v=const}$ where $\epsilon$ denotes the energy of a molecule and *T* its temperature. This energy can be further decomposed into different parts, such that $\epsilon = \epsilon ^{Tr}+\epsilon ^R+\epsilon ^V+\epsilon ^E$. Each part is associated with a different degree of freedom of the system. Here, *Tr* stands for translational, *R* for rotational, *V* for vibrational and *E* for the electronic contributions to the total energy of the system (Atkins and de Paula 2010; Tinoco 2002). With this decomposition in mind, the different archetypal molecules associated with a particular heat capacity are compared in Fig. 10. In both panels of that figure, the rows correspond to the three molecules in the QM9 data set (test set) that have been mapped closest to a vertex of the latent simplex and have thus been identified as being extremes with respect to the heat capacity. Out of a total of 20 vertices, molecules in close proximity to four of them are displayed here. Panel 10a shows the configuration of six archetypal molecules. The upper three are all associated with a low heat capacity while the lower three all have a high heat capacity. This result can easily be interpreted, as the lower heat capacity can be traced back to the shorter chain length and the higher number of double bonds of these molecules, which makes them more stable and results in a lower vibrational energy *V* and subsequently in a lower heat capacity. The inverse is observed for the linear archetypal molecules with higher heat capacities, which show, relative to their size, a lower number of double bonds and a long linear structure. Panel 10b shows both linear (lower row) and non-linear archetypal molecules (upper row) but with similar atomic mass. Here, the non-linear molecules containing a cyclic structure in their geometry, are more stable and therefore have an overall slightly lower heat capacity compared to their linear counterparts of the same weight, shown in the second row.

*Experiment 3: Interpolation Between Two Archetypal Molecules.* Interpolation is performed by plotting the samples from the test set which are closest to the connecting line between the two archetypes. As a result, one can observe a smooth transition from a molecule with a ring structure to a linear chain molecule. Both the starting and the end point of this interpolation is characterized by a similar heat capacity, such that these archetypes differ only in their geometric configuration but not with respect to their side information. As a consequence, any molecule in close proximity to that connecting line can differ only with respect to its structure, but *must* display a similarly high heat capacity. Figure 11 shows an example of such an interpolation.

*Experiment 4: The Role of Side Information and the Exploration of Chemical Space.* Deep AA structures latent spaces both according to the information contained in the input to the encoder as well as the side information provided. As a consequence, any molecule characterized as a *true* mixture of two or more archetypes, given a specific side information such as *heat capacity*, might suddenly be identified as archetypal should the side information change accordingly. In the following, archetypal molecules with respect to *heat capacity* as the side information are compared to archetypes obtained while providing the *band gap energy* of each molecule as the side information. In Fig. 12a archetypal molecules with both the highest and the lowest heat capacities are displayed while 12b shows archetypes with highest and lowest band gap energies. The archetypes significantly differ in their structure as well as their atomic composition. For example, archetypal molecules with low heat capacity are rather small, with only few C and O atoms, while archetypal molecules with a low band gap energy are characterized by ring structures containing N and H atoms. This illustrates the essential role of side information for learning and subsequently enabling the interpretation of the latent representation.

### Alternative Priors For Deep Archetypal Analysis

The standard normal distribution is a common choice for the prior distribution $p(\mathbf {t})$ due to its simplicity and closed form expression for the KL divergence. However, alternative priors might influence the inferred archetypes or prove beneficial when learning the structure of the latent space. Leaving aside the wide range of well explored priors for vanilla VAEs, we explore a hierarchical prior that directly corresponds to the generative model of linear AA presented in Eq. 5, i.e. isotropic Gaussian noise around a linear combination of the archetypes:

16 $$\begin{aligned} \mathbf {m} \sim \text {Dir}_k(\varvec{\alpha }=\mathbf {1}) \quad \wedge \quad \mathbf {t} \sim \mathcal {N}(\mathbf {m}Z^{\text {fixed}}, \mathbf {I}) \end{aligned}$$

The estimation of the KL divergence given in Eq. 8 is based on Monte-Carlo sampling. In order to qualitatively compare the standard normal prior and the sampling Dirichlet prior, we train the respective deep AA model on the JAFFE data set with $k=4$ archetypes, implying a 3-dimensional latent space. The architecture used is similar to the previous experiments but we additionally *learn* the variance of the decoder. The Lagrange parameters or weights in Eq. 15 are set to 1e3 for the archetype loss and to 1e2 for the KL divergence.

**Fig. 13:**
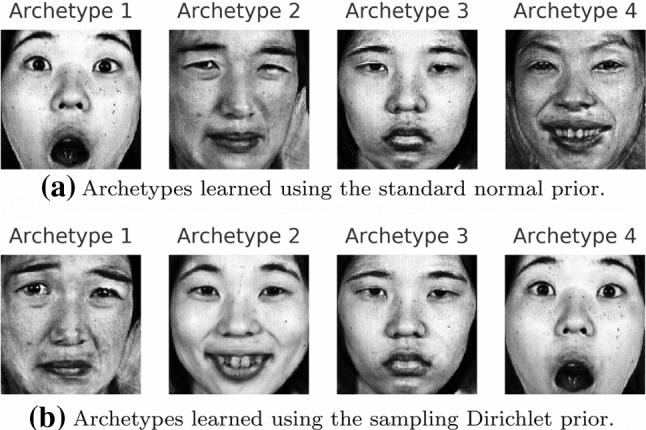
Deep AA with $k = 4$ archetypes using two different priors, which both identify similar archetypes

**Fig. 14:**
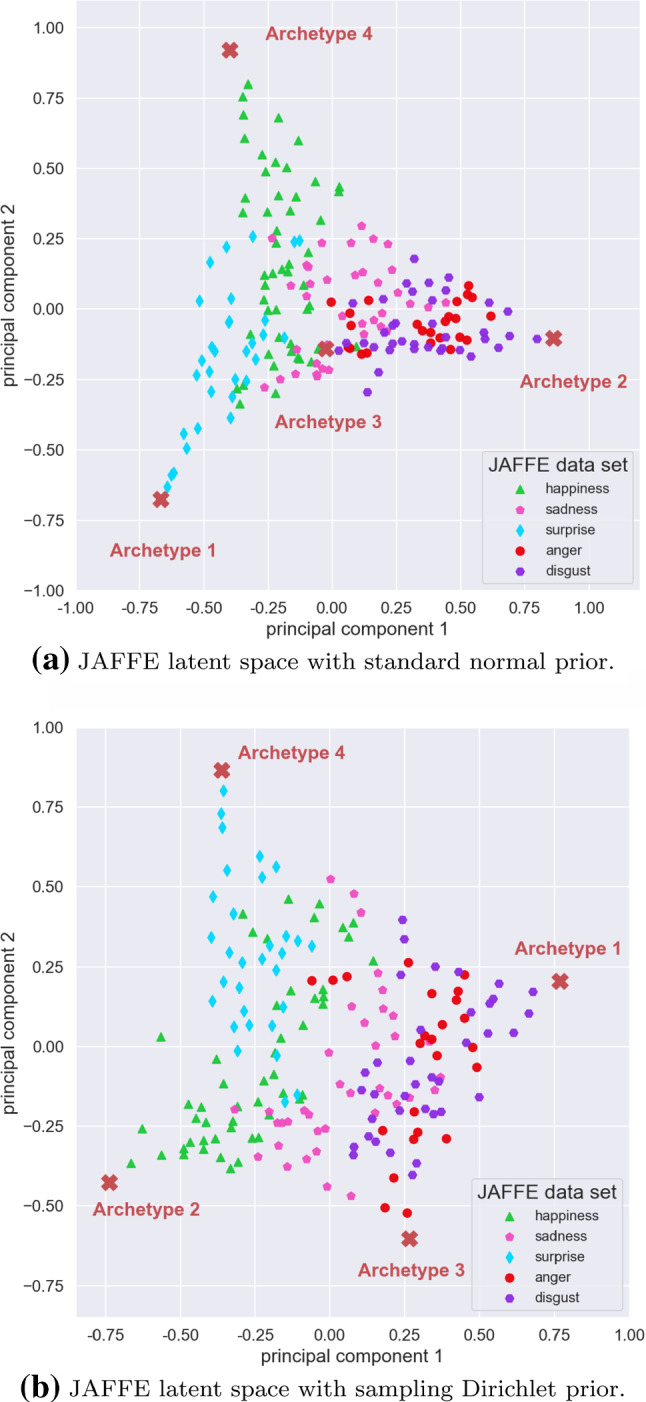
Latent spaces for the two different priors projected onto the first two principal components. The explained variances are: **a** 0.74 and **b** 0.757

Finally, Fig. 13 shows examples of the inferred archetypes for the standard normal prior (upper row) and the sampling Dirichlet prior (lower row). In conclusion, different priors do not seem to strongly affect the inferred archetypes. However, the structure of latent spaces do differ, which can be seen when projecting them onto the first two principal components as shown in Fig. 14. As a reference, a uniformly filled simplex would result in a triangular shaped projection. The difference seen here is caused by large gaps in the higher-dimensional simplex when using the hierarchical prior, which we assume is mainly due to the high variance estimation of the KL divergence.

In our experience, the choice of the prior is not of primary concern for finding meaningful archetypes, as long as it encourages the latent space to be spread out inside the simplex, be that via a standard normal, a uniform or—as in this case—a hierarchical prior.

## Practical Considerations for Using Deep AA

In the following, we provide general aspects worth considering when deciding whether the use of deep AA might be appropriate, given a specific data set.

Linear Archetypal Analysis relies on the additivity assumption, as data points are described as a weighted sum of the archetypes, with the weights constrained to be non-negative. This mixing procedure is performed directly on the input data. In deep AA, on the other hand, the mixing is performed only on the latent representation of the input data. This allows more flexibility regarding the type of input data, e.g. text, images etc., but it also relaxes the additivity assumption, as the encoder *learns* a representation on which this assumption is (approximately) valid. Nevertheless, for the interpretation of the archetypes, convex mixing should *a priori* be a justifiable assumption. In general, for data without explicit cluster structure (deep) AA poses an interesting possibility. On the ten digits in MNIST for example, a clustering might be more appropriate while deep AA could provide additional insight when applied on a single digit class. The goal of deep AA is to optimize for the most compact latent code such that interpretability of the archetypes—on a qualitative level—remains possible. Having too many archetypes would likely obfuscate the meaning of an individual archetype. Furthermore, as deep AA relies on the latent simplex as a geometrical structure, Euclidean distances need to be meaningful with respect to the dimensionality of the latent space, generally encouraging l*ow* dimensionality. But this is of course true for all flavors of AA. In practice, inspecting the local data density in the neighborhood of the inferred archetypes in latent space is an important post-processing step. If an archetype appears to have no latent samples close to it, it might be considered an outlier.

## Conclusion

We have presented in this paper an extension of linear Archetypal Analysis, a technique for exploratory data analysis and interpretable machine learning. By performing Archetypal Analysis in the latent space of a deep information bottleneck, we have demonstrated that the learned representation can be structured in a way that allows it to be characterized by its most extremal or archetypal representatives. As a result, each observation in the data set can be described as a convex mixture of these extremes. Endowed with such a structure, a latent space can be explored by varying the mixture coefficients with respect to the archetypes, instead of exploring the space by uniform sampling. Furthermore, we have demonstrated the need for including side information into the process of learning latent archetypal representations. Extremeness can only be understood with respect to a given property. Therefore, providing such a property through side information is essential in order to learn interpretable latent archetypes. In contrast to the original archetype model, our method offers three advantages: First, our model learns representations in a data-driven fashion, thereby reducing the need for expert knowledge. Second, our model can learn appropriate transformations to obtain meaningful archetypes, even if non-linear relations between features exist. Third, the incorporation of side information. The application of this new method is demonstrated on a sentiment analysis task, where emotion archetypes are identified based on female facial expressions, for which multi-rater based emotion scores are available as side information. A second application illustrates the exploration of the chemical space of small organic molecules and demonstrated how crucial side information is for interpreting the geometric configuration of these molecules.

## Electronic supplementary material

Below is the link to the electronic supplementary material.

Supplementary material 1 (pdf 13558 KB)

## Footnotes

1 Note that $i=1..m$ (and not up to *n*), which reflects that deep neural networks usually require batch-wise training with batch size *m*.

2 The code is available via https://github.com/bmda-unibas/DeepArchetypeAnalysis
