# Supplementary material for: Learning Extremal Representations with Deep Archetypal Analysis
Source: Int J Comput Vis. 2020 Dec 23;129(4):805–20. doi: 10.1007/s11263-020-01390-3 (PMC8550171; doi:10.1007/s11263-020-01390-3)
Supplement: Supplementary file 1 — Supplementary material 1 (pdf 13558 KB) [file 11263_2020_1390_MOESM1_ESM.pdf]

International Journal of Computer Vision IJCV

SUPPLEMENTARY MATERIAL

Learning Extremal Representations with Deep Archetypal Analysis

Sebastian Mathias Keller[1,2], Maxim Samarin[1,3], Fabricio Arend Torres[1], Mario Wieser[1], and Volker Roth[1]

[1]University of Basel

S1 Stability of Inferred Archetypes: Bootstrapping Experiment

In order to demonstrate the stability of the inferred archetypes with respect to their interpretation, we evaluate our method on 40 distinct sets of bootstrap samples of the JAFFE data set. The general setup is similar to section ??. The weights of the archetype and side information loss are 1e2 and 2e2, respectively, while the weight of the reconstruction loss is set to 0.4. The weight of the KL divergence is initialized with 5e3 and then slowly decreased until it reaches the target weight of 4e1.
Figures S1 and S2 show the true input images that were mapped closest to the vertices of the latent simplex when mapping the whole data set (i. e. including the bootstrap hold-out set) into the latent space at test time. The scatter plots also show the latent distribution of the whole data set. Colors indicate the *argmax* of the five emotion scores, which were used as side information during training. We can see that the inferred archetypes – here: the closest true input images – consist of three distinct "extreme" emotions for the majority of the runs. Importantly, some images are recognized as archetypal through multiple runs even though the training data sets had different compositions each time. Figure S3 shows the distribution of the different combinations of inferred archetypal emotions. We note that the predominant combination contains the emotions "surprised", "happy" and "angry".

[2]Corresponding author: `sebastianmathias.keller@unibas.ch`
[3]Corresponding author: `maxim.samarin@unibas.ch`

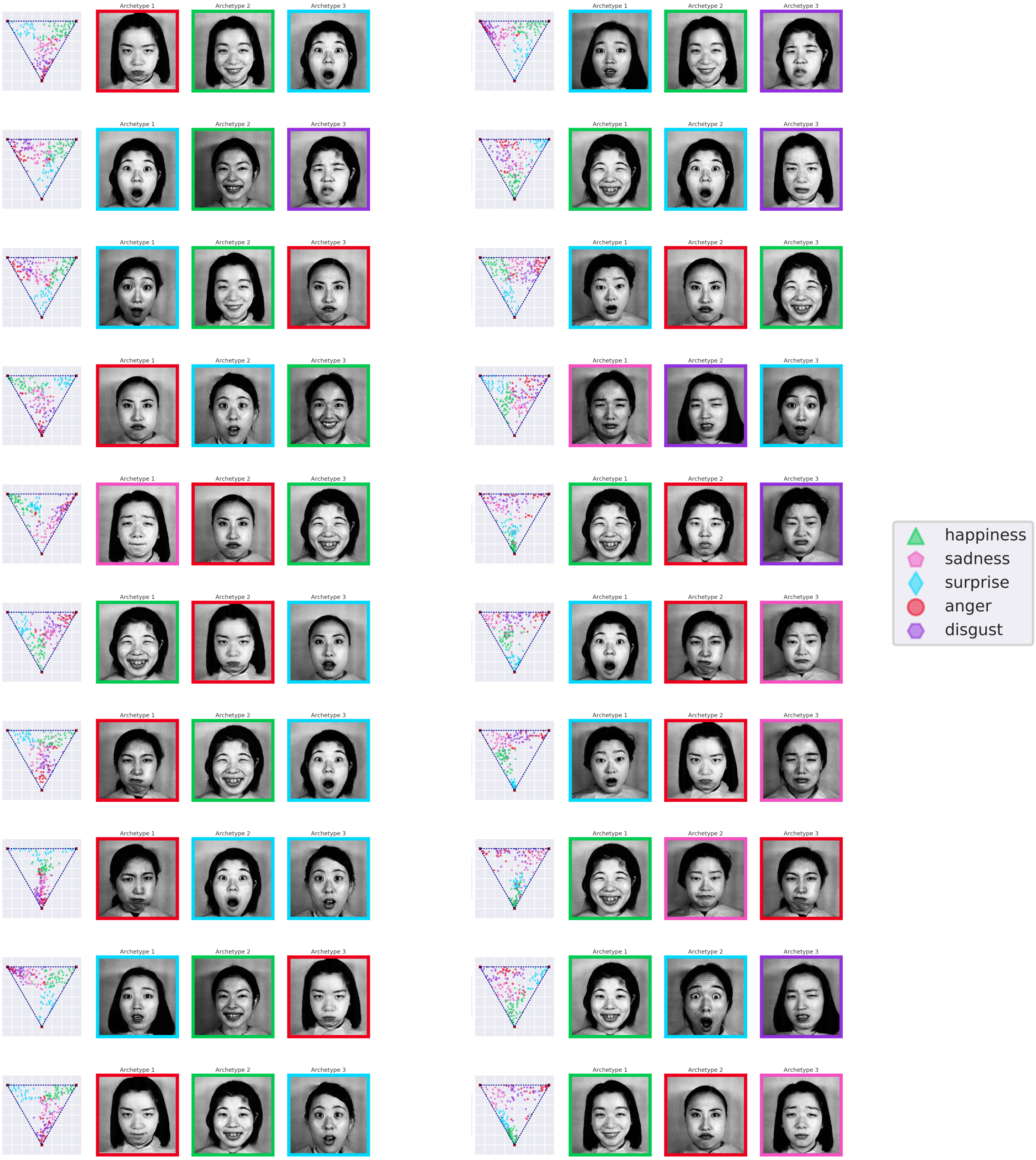

Figure S1: JAFFE archetypes and latent distribution trained on 20 distinct bootstrap data sets.

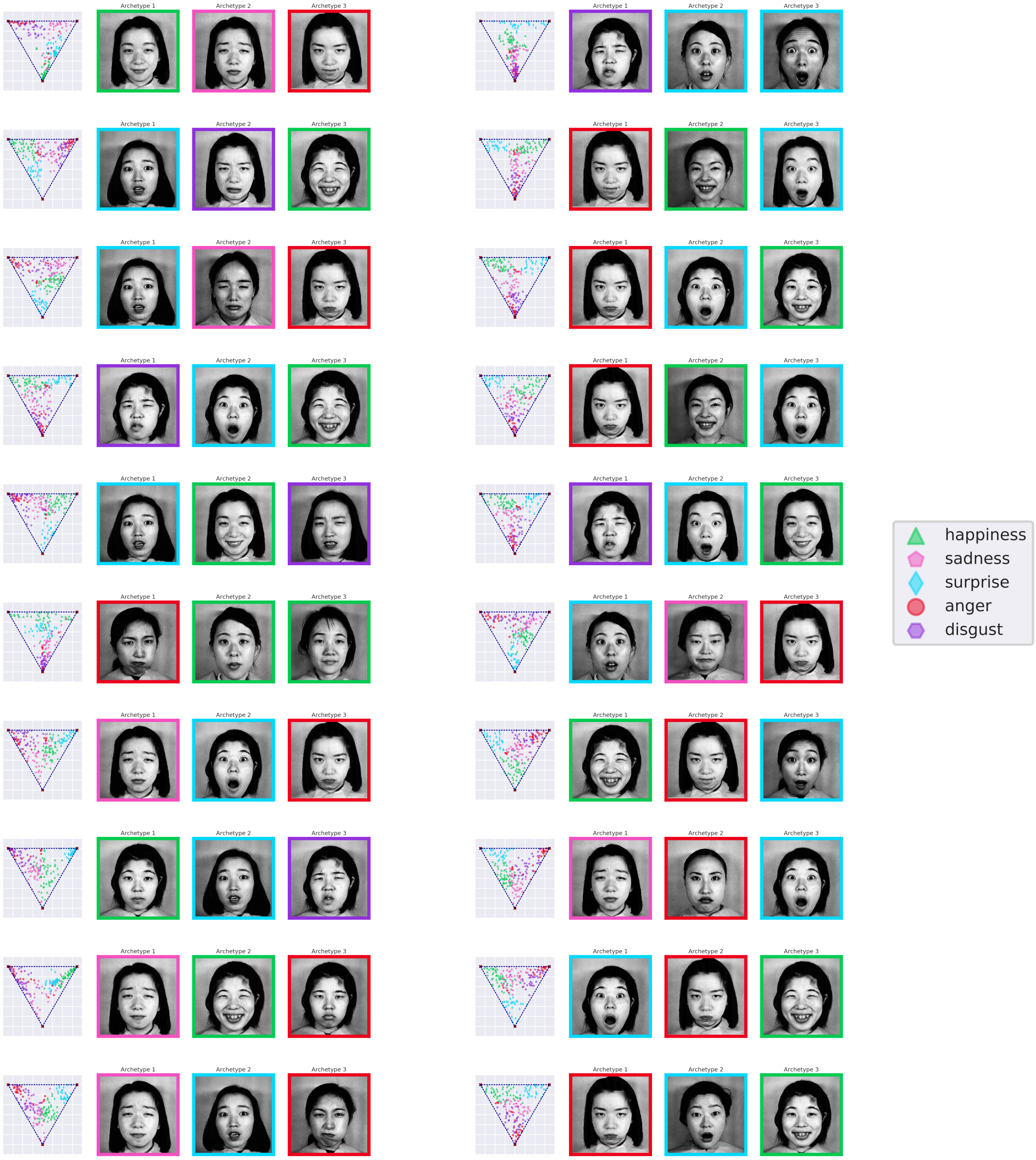

Figure S2: JAFFE archetypes and latent distribution trained on 20 distinct bootstrap data sets.

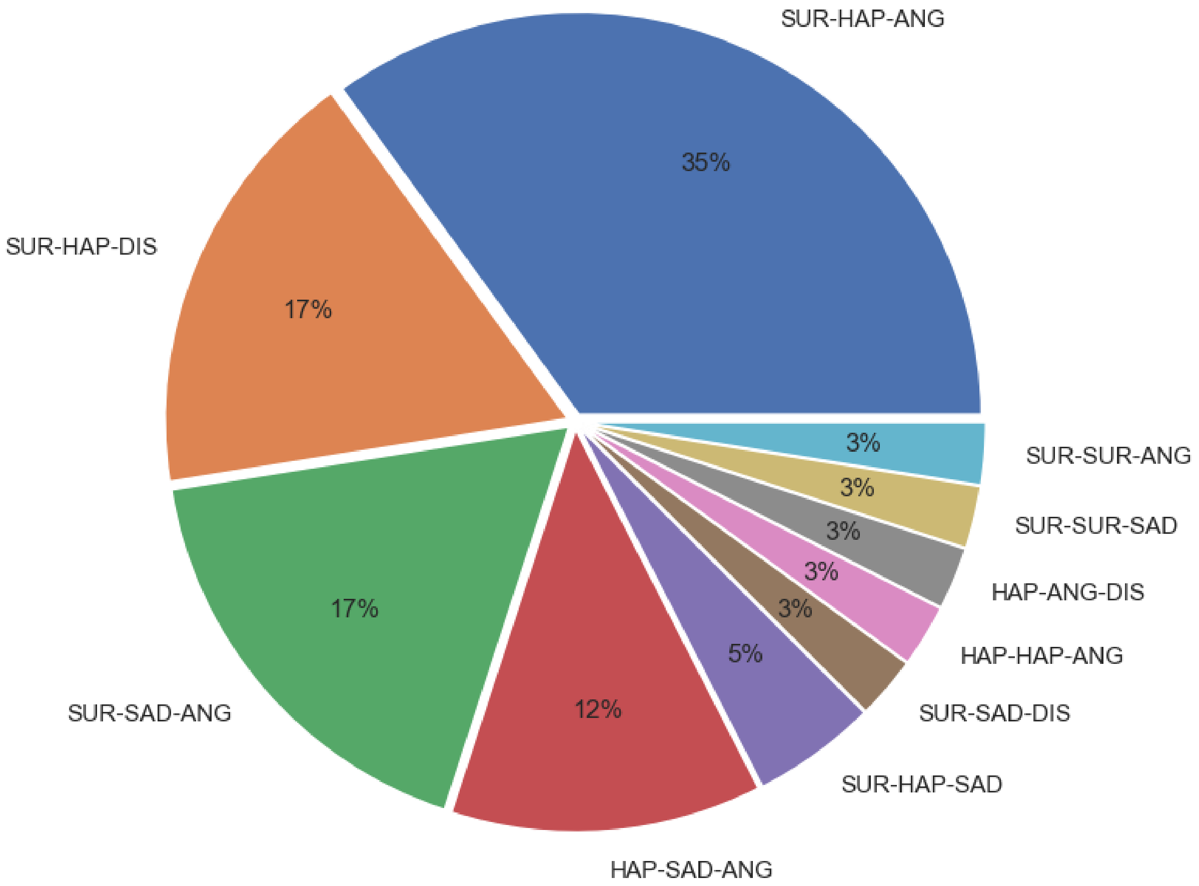

Figure S3: Distribution of combinations of inferred archetypal emotions based on 40 bootstrap runs.
